# Comparison of two portable metabolic systems for measuring energy expenditure at rest and during exercise in untrained women

**DOI:** 10.3389/fphys.2025.1583703

**Published:** 2025-07-01

**Authors:** Feng Gao, Yujing Chan-Yu, Zhi Li, Yongfu Liu, Fei Liu, Di Liu, Wenjun Yu, Weiwei Chen, Junhua Wang, Shenglong Le

**Affiliations:** ^1^ Department of Physical Therapy, Taihe Hospital, Hubei University of Medicine, Shiyan, China; ^2^ School of Biomedical Engineering, Hubei University of Medicine, Shiyan, China; ^3^ Center for Diabetes Rehabilitation Research, Taihe Hospital, Hubei University of Medicine, Shiyan, China; ^4^ Department of Rehabilitation, Taihe Hospital, Hubei University of Medicine, Shiyan, China; ^5^ Department of Physical Education, Shanghai Jiao Tong University, Shanghai, China

**Keywords:** COSMED K5, CORTEX METAMAX 3B, energy expenditure, indirect calorimetry, metabolic measurement, wearable metabolic systems

## Abstract

**Purpose:**

Portable metabolic systems are used as the “gold standard” for measuring energy expenditure (EE) in the development and validation of wearable devices. This study aimed to compare EE measurements obtained using the COSMED K5 (K5) and CORTEX METAMAX 3B (M3B) during the resting state and submaximal-intensity exercise in women without self-reported regular exercise training.

**Methods:**

Twenty women aged 21.4 ± 1.5 years completed two measurements, including resting in a seated position and cycling on a simple upright ergometer at 30 W, 40 W, 50 W, and 60 W. Average EE and other metabolic parameters were compared between K5 and M3B. Differences between K5 and M3B were assessed using the paired-samples t-test, and the effect size was calculated as Cohen’s d. Agreement between the two systems was evaluated by calculating Pearson correlation coefficients and visually examining Bland–Altman plots.

**Results:**

The number of participants who completed resting and exercise measurements was 18 and 19, respectively. For resting EE, the mean values measured using K5 were 33.4% higher than those measured using M3B (*p* < 0.001, Cohen’s d = 1.47). Similar differences were observed for cycling at 30 W (15.8%, *p* < 0.001, Cohen’s d = 1.50), 40 W (16.1%, *p* < 0.001, Cohen’s d = 1.68), 50 W (14.8%, *p* < 0.001, Cohen’s d = 1.28), and 60 W (14.6%, *p* < 0.001, Cohen’s d = 1.29). Pearson correlation coefficients between EE measured using K5 and M3B was 0.66 for 30 W cycling (*p* = 0.002) and 0.62 for 40 W cycling (*p* = 0.005).

**Conclusion:**

K5 and M3B show significant differences in EE measurements during rest and exercise among untrained female individuals, indicating systematic bias in EE measurement between the two systems. Thus, careful consideration is essential when interpreting the results of wearable device studies that use different automated metabolic systems.

## 1 Introduction

Energy expenditure (EE) has emerged as the most important indicator of physical activity monitored by wearable devices. For the population with metabolic risk factors, especially those with metabolic disorders such as obesity and diabetes, the measurement of daily EE can provide fundamental information for their effective health management (Hill et al., 2012; Caron et al., 2016). Indirect calorimetry is a well-accepted method of evaluating EE by gauging the oxygen consumed (VO_2_) and the carbon dioxide released (VCO_2_) (Haugen et al., 2007). Portable systems, such as COSMED K5 (K5) and CORTEX METAMAX 3B (M3B), have facilitated these measurements (Macfarlane, 2017; Overstreet et al., 2017). Compared to the Douglas bag method, K5 has demonstrated validity for measuring VO_2_ during rest and cycling at intensities ranging from 50 to 250 W with a mean error of less than 5%, although its breath-by-breath mode underestimated VCO_2_ by up to 9% and the respiratory exchange ratio (RER) by 0.09 at workloads ≥150 W (Crouter et al., 2019). M3B has been reported to produce acceptably stable (<2% error) and reliable (<2.5% error) measurements, with validity against the Douglas bag method showing accurate resting values (VO_2_: −0.3%, VCO_2_: +1.1%) but overestimating VO_2_/VCO_2_ by 10%–12% during moderate cycling and by 14%–17% during vigorous cycling (Macfarlane and Wong, 2012).

Some previous studies have investigated the agreement of results between different portable metabolic systems (Leprêtre et al., 2012; Van Hooren et al., 2024). The agreement between COSMED K4b2 and CORTEX METAMAX II was poor for VCO_2_ (20.3% bias) and RER (−18.9% bias) during a graded exercise cycle test in trained male cyclists, while it was acceptable for ventilation (VE) and VO_2_ (Leprêtre et al., 2012). More recently, a cross-comparison study of 15 metabolic systems, including K5 and M3B, showed that the absolute error of VO_2_ (1.10%–13.3%), VCO_2_ (1.07%–18.3%), RER (0.62%–14.8%), and EE (0.59%–12.1%) exhibited considerable differences between the systems during the simulations (Van Hooren et al., 2024). Notably, during cycling at maximal steady-state intensity by three well-trained, healthy individuals, the relative differences between systems mostly, but not always, matched those observed during the simulations (Van Hooren et al., 2024). Collectively, these findings highlight the critical need for further comparison of different portable metabolic systems, especially in human exercise contexts.

Women are more likely to use wearable devices than men (Chandrasekaran et al., 2020). Studies indicate that nearly half (48.94%) of female university students currently use such wearable devices, with 65.14% of non-users expressing future adoption intent (Shin et al., 2023). Moreover, these devices have been shown to effectively increase physical activity levels and aid in weight management, empowering them to manage health, particularly for individuals not currently meeting physical activity guidelines (Brickwood et al., 2019; Ellingson et al., 2019; Nuss et al., 2021; AlSwayied et al., 2022; Ferguson et al., 2022). The accuracy of these measurements is crucial for perceived usefulness, user adoption, long-term adherence, and achieving health benefits (Lindgren et al., 2019; Hu et al., 2020; El-Gayar and Elnoshokaty, 2023). K5 and M3B have often been used as the criterion methods to develop prediction equations and assess the accuracy of EE estimated by wearable devices (Macfarlane, 2017; O’Driscoll et al., 2020; Chevance et al., 2022; Huang et al., 2024). Unfortunately, limited information is available regarding the comparison of measurements obtained from these two portable metabolic systems, especially when applied to female individuals with relatively low physical activity and physiological levels. Thus, to address this gap in the literature and provide a more accurate assessment for untrained female individuals, the objective of this study was to compare EE measured by K5 and M3B during rest and exercise in Chinese women without self-reported regular exercise training and to evaluate the degree of agreement between these portable metabolic units in EE measurement.

## 2 Materials and methods

### 2.1 Participants

Twenty healthy Chinese female individuals were recruited from a local medical university (Table 1). Participants were screened based on the inclusion and exclusion criteria using a lifestyle and disease questionnaire and ACSM risk stratification, which included medical history, signs and symptoms, and risk factors (Jonas and Phillips, 2012). The inclusion criteria consisted of healthy young female individuals aged 18–30 years who reported engaging in no more than three weekly exercise sessions. The exclusion criteria included pregnancy and any acute illness (e.g., flu, fever, or infection) within the past 2 weeks.

**TABLE 1:** Physical characteristics of female participants (n = 20).

Age (year)	Height (cm)	Weight (kg)	BMI (kg/m^2^)	BF (%)
21.4 ± 1.5	162.2 ± 5.1	55.8 ± 7.8	21.2 ± 2.7	31.6 ± 5.9

BMI, body mass index; BF, body fat percentage.

The study was conducted in accordance with the Declaration of Helsinki and approved by the Ethics Committee of the researchers’ institution (2022KS041). All participants were informed of the purpose, procedures, and potential risks of the study, and they provided written informed consent prior to their first visit, which was conducted to familiarize them with the devices.

### 2.2 Study design

Data for the comparison were collected during two identical measurement procedures, one using K5 and the other using M3B, with a time interval of 3–7 days between the two measurements. Participants completed these two measurements randomly but at the same time of day and with the same athletic clothes and shoes to avoid the influence of circadian rhythm variance on metabolic responses (Thun et al., 2015). Additionally, all participants completed two measurements within 2 weeks, specifically within the first 5 days after the onset of menstruation and before the next onset, to minimize the effects of the menstrual cycle (Janse et al., 2019). Participants were instructed to consume only a light diet and avoid any vigorous physical activity, smoking, caffeine consumption, or alcohol intake for 12 h prior to measurements. They were also asked not to consume any food or drink (except water) at least 3 h prior to measurements.

**FIGURE 1:**
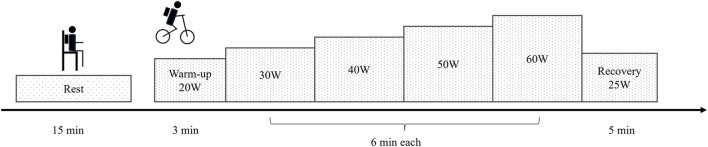
Measurement protocol.

The measurement consisted of two sessions, resting and exercise, conducted in an air-conditioned room (Figure 1). After being equipped with K5 or M3B, the participant sat on a chair for 15 min without any task to measure EE during rest. Following the resting measurement, the participant performed incremental cycling on a simple upright electronically braked ergometer (Monark 839 E, Monark, Vansbro, Sweden). After a 3-min warm-up at 20 W, each participant performed a 6-min stage-incremental exercise starting at 30 W and increasing by 10 W up to 60 W, followed by a 5-min recovery period at 25 W. The pedaling frequency was maintained at 60 rpm (Jacobs et al., 2013). For each session, ambient temperature, relative humidity, and barometric pressure were recorded.

More than 2 days prior to the first measurement, participants performed a 30-min practice session on the ergometer using all devices to familiarize themselves with the cycling process and equipment. The seat and handlebar heights based on participants’ personal preferences were recorded. Meanwhile, a wall-mounted height scale accurate to the nearest 0.1 cm was used to measure their heights. The InBody 770 bioimpedance device (Biospace Co., Ltd., Seoul, Korea) was used to measure the body mass and body composition of the participants.

### 2.3 Portable metabolic systems

K5 (COSMED, Rome, Italy) has been described in detail by Crouter et al. (2019). In brief, it is designed as a single unit and is portable, with a total weight of 900 g, and it is worn on the participant’s back. Gas exchange data were collected in the breath-by-breath mode using a bidirectional digital turbine connected to a rubberized facemask; the data were transmitted via Bluetooth to *OMNIA software* 2.4.2 for visualization and additional storage.

M3B (CORTEX Medical, Leipzig, Germany) has been described in detail by Vogler et al. (2010). In brief, it contains two parts and is portable with a total weight of 580 g, and it is worn on the participant’s chest. Gas exchange data were collected in the breath-by-breath mode using a bidirectional digital turbine connected to a rubberized facemask; the data were transmitted via Bluetooth to *MetaSoft Studio software* for visualization and additional storage.

Prior to each measurement, both metabolic systems were calibrated in compliance with the manufacturer’s instructions. The heart rate belt used was Polar H10 (Polar Oy, Kempele, Finland).

### 2.4 Sample size

The sample size was estimated using G*Power version 3.1.9.6 (Franz Faul, University of Kiel, Germany) with a power of 0.9 at a significance level of 0.05 for the paired-samples t-test. Based on calculations using data from the previous study (Leprêtre et al., 2012), 14 participants were required. Considering potential dropouts, 20 participants were recruited.

### 2.5 Statistical analysis

EE, metabolic equivalents (METs), VO_2_, VCO_2_, VE, and RER were extracted and analyzed during rest and each cycling stage. Values for analysis were averaged between 5:00 and 13:00 min during rest and between 1:30 and 5:30 min for each cycling stage. The normality of the data was tested using the Shapiro–Wilk test. Differences in the metabolic variables measured by K5 and M3B were assessed using the paired-samples t-test. If a significant difference was found, Cohen’s d was calculated to assess the effect size, with thresholds defined as follows: 0.2, trivial; 0.6, small; 1.2, moderate; 2.0, large; 4.0, very large; and ≥4.0, extremely large (Hopkins et al., 2009; Liu et al., 2022). Agreement between the two systems was evaluated using Pearson correlation coefficients and Bland–Altman plots. Pearson correlation coefficients were categorized as follows: moderate (0.40–0.69), strong (0.70–0.89), and very strong (0.90–1.00) (Deysel et al., 2024). All statistical analyses were performed using RStudio (version 2024.12.0) software. The values measured by K5 and M3B were presented as the mean ± standard deviation (SD). Statistical significance was predetermined at *p* < 0.05.

## 3 Results

### 3.1 Participants’ characteristics

The descriptive characteristics of the participants are presented in Table 1. Participants were generally healthy. Eighteen participants completed resting measurements, while 19 completed exercise measurements. No significant differences were observed between K5 and M3B for laboratory temperature (23.7 ± 0.9 vs 23.4°C ± 1.1°C; *p* = 0.20), barometric pressure (736.6 ± 2.2 vs 735.5 ± 2.1 mmHg; *p* = 0.08), and relative humidity (69.9% ± 3.6% vs 71.1% ± 3.6%; *p* = 0.28).

### 3.2 Metabolic parameters during resting

**FIGURE 2:**
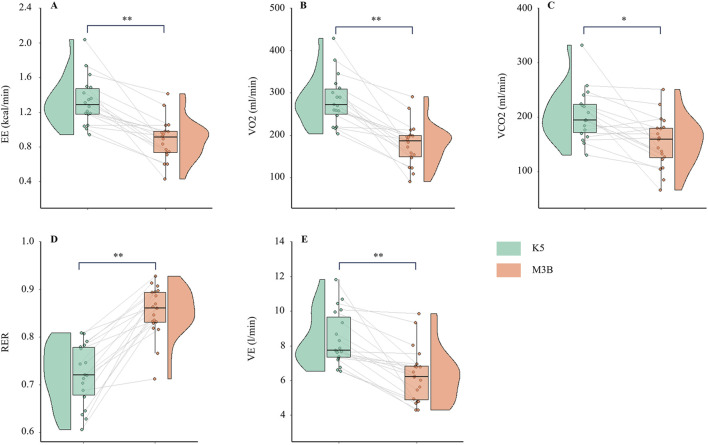
Comparison of metabolic variables at rest between K5 and M3B (N = 18). **(A)** Results of EE, **(B)** results of VO_2_, **(C)** results of VCO_2_, **(D)** results of VE, and **(E)** results of RER. K5, COSMED K5; M3B, CORTEX METAMAX 3B; EE, energy expenditure; VO_2_, oxygen consumed; VCO_2_, carbon dioxide released; VE, ventilation; RER, respiratory exchange ratio. *P < 0.01, **P < 0.001.

**FIGURE 3:**
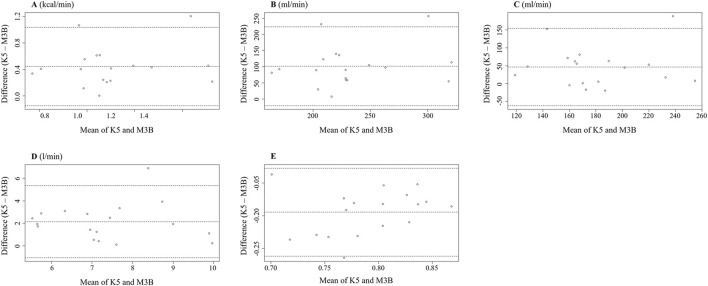
Bland–Altman plot measured using K5 and M3B at rest (N = 18). **(A)** Results of EE, **(B)** results of VO_2_, **(C)** results of VCO_2_, **(D)** results of VE, and **(E)** results of RER. K5, COSMED K5; M3B, CORTEX METAMAX 3B.

The metabolic parameters during resting were compared between K5 and M3B using the paired-samples t-test, as shown in Figure 2. The mean values measured by K5 were significantly higher for EE than those measured by M3B (1.33 ± 0.28 vs 0.88 ± 0.24 kcal/min, t = 6.237, *p* < 0.001, Cohen’s *d* = 1.47; Figure 2A). Similar results were observed for VO_2_ (t = 6.904, *p* < 0.001, Cohen’s *d* = 1.63; Figure 2B), VCO_2_ (t = 3.550, *p* = 0.002, Cohen’s *d* = 0.84; Figure 2C), and VE (t = 5.559, *p* < 0.001, Cohen’s *d* = 1.31; Figure 2D). However, the mean values for RER were significantly lower for K5 than for M3B (t = −8.579, *p* < 0.001, Cohen’s *d* = 2.02; Figure 2E). Pearson correlation analysis revealed a moderate and significant correlation only for VE measured using the two devices (r = 0.481, *p* = 0.04; Table 2). In the Bland–Altman plots, the percentage of values within the limits of agreement ranged from 89.5% to 94.7% (Figure 3).

**TABLE 2:** Correlations among measurements of metabolic parameters during rest and cycling between K5 and M3B (n = 19).

Metabolic parameters	Resting^a^	30 W	40 W	50 W	60 W
Energy expenditure (kcal/min)	0.329	0.660^b^	0.621^b^	0.404	0.331
Metabolic equivalents	—	0.731^b^	0.772^b^	0.673^b^	0.641^b^
Oxygen consumed (mL/min)	0.341	0.653^b^	0.614^b^	0.401	0.362
Carbon dioxide released (mL/min)	0.284	0.690^b^	0.726^b^	0.618^b^	0.472^c^
Ventilation (L/min)	0.481^c^	0.714^c^	0.857^b^	0.794^b^	0.585^b^
Respiratory exchange ratio	0.282	0.581^b^	0.533^c^	0.793^b^	0.767^b^

K5, COSMED K5; M3B, CORTEX METAMAX 3B.

^a^ n = 18.

^c^ *P* < 0.05.

^b^ *P* < 0.01.

### 3.3 Metabolic parameters during exercise

**FIGURE 4:**
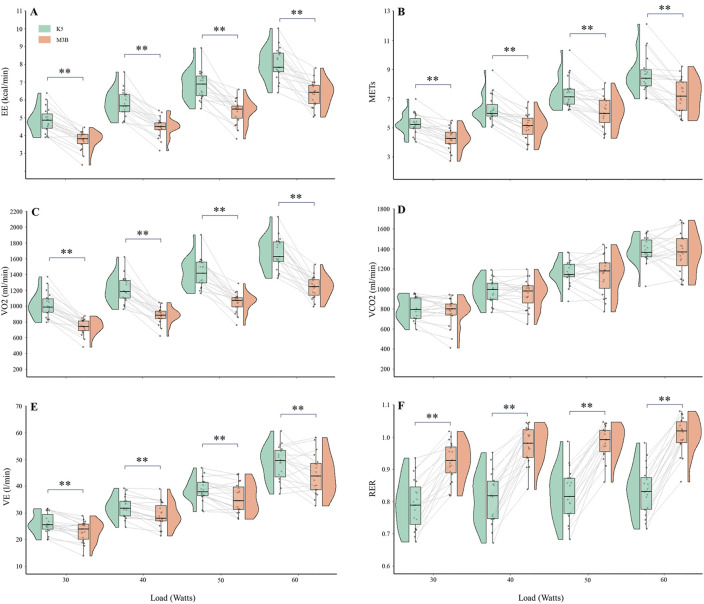
Comparison of metabolic variables during cycling at several loads between K5 and M3B (N = 19). **(A)** Results of EE, **(B)** results of METs, **(C)** results of VO_2_, **(D)** results of VCO_2_, **(E)** results of VE, and **(F)** results of RER. K5, COSMED K5; M3B, CORTEX METAMAX 3B; EE, energy expenditure; METs, metabolic equivalents; VO_2_, oxygen consumed; VCO_2_, carbon dioxide released; VE, ventilation; RER, respiratory exchange ratio. *P < 0.01 and **P < 0.001.

**FIGURE 5:**
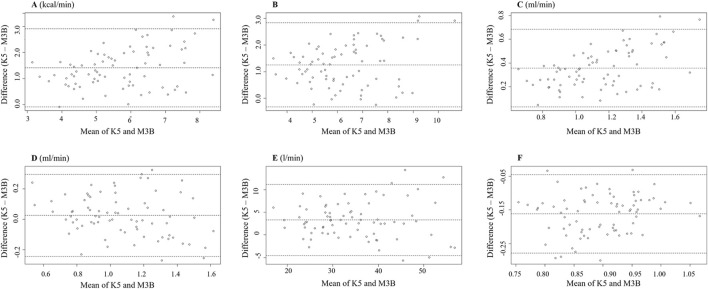
Bland–Altman plot measured using K5 and M3B during cycling (n = 19). **(A)** Results of EE, **(B)** results of METs, **(C)** results of VO_2_, **(D)** results of VCO_2_, **(E)** results of VE, and **(F)** results of RER. K5, COSMED K5; M3B, CORTEX METAMAX 3B.

The metabolic parameters during cycling at 30 W, 40 W, 50 W, and 60 W were compared between K5 and M3B using the paired-samples t-test, as shown in Figure 4. Significant mean differences were found for EE, METs, VO_2_, VE, and RER across all loads, while no significant difference was found for VCO_2_. The mean values of EE measured by K5 were all significantly higher than those measured by M3B for 30 W (4.85 ± 0.71 vs 3.72 ± 0.52 kcal/min, t = 9.201, *p* < 0.001, Cohen’s *d* = 1.50), 40 W (5.81 ± 0.75 vs 4.48 ± 0.55 kcal/min, t = 9.768, *p* < 0.001, Cohen’s *d* = 1.68), 50 W (6.82 ± 0.82 vs 5.35 ± 0.68 kcal/min, t = 7.769, *p* < 0.001, Cohen’s *d* = 1.28), and 60 W (8.05 ± 0.90 vs 6.34 ± 0.74 kcal/min, t = 7.820, *p* < 0.001, Cohen’s *d* = 1.29) (Figure 4A). In addition, similar results were found for METs (t = 6.244–8.567, all *p* < 0.001, Cohen’s *d* = 1.43–1.97; Figure 4B), VO_2_ (t = 9.262–11.100, all *p* < 0.0.001, Cohen’s *d* = 2.12–2.55; Figure 4C), and VE (t = 2.999–5.416, *p* = 0.008–0.000, Cohen’s *d* = 0.69–1.24; Figure 4E). However, the mean values for RER were significantly lower for K5 than for M3B (t = −16.394–8.976, all *p* < 0.001, Cohen’s *d* = 2.06–3.76; Figure 4F). Pearson correlation coefficients were mostly moderate (r = 0.40–0.69) or high (r = 0.70–0.89) for the corresponding metabolic variables measured by K5 and M3B (Table 2). However, no significant relationship was observed for EE at 60 W or for VO_2_ at 50 W and 60 W. In the Bland–Altman plots of all data at different loads, the percentage of values within the limits of agreement were 97.4% for EE and VO_2_, 96.1% for METs and VCO_2_, and 93.4% for VE and RER (Figure 5).

## 4 Discussion

To our knowledge, this study represents the first attempt to assess the consistency of EE measurements between two portable metabolic systems (K5 and M3B) in untrained female individuals under resting and exercising conditions. The research findings demonstrated that EE measurements differed significantly between K5 and M3B. Furthermore, there were only moderate correlations between K5 and M3B in measuring EE during rest and exercise.

K5 and M3B represent the latest generation of portable metabolic measurement systems, offering breath-by-breath technology that enables high-precision and reliable measurements of VO_2_ and VCO_2_. These devices are engineered for portability and user comfort, facilitating ease of wear while providing real-time metabolic data across diverse exercise intensities and durations (Macfarlane, 2017). Consequently, the K5 and M3B systems are increasingly adopted as criterion measures in both developing EE prediction algorithms and validating their ecological validity for wearable device applications (Macfarlane, 2017; O’Driscoll et al., 2020; Chevance et al., 2022; Huang et al., 2024). However, few studies have validated the accuracy of EE measurements using the M3B and K5 as most existing research has focused on respiratory gas variables such as VO_2_ and VCO_2_. Perez-Suarez et al. (2018) reported that K5 accurately measured EE under low-intensity cycling (60 W) and resting conditions but underestimated EE by 6.6% during moderate-intensity cycling (130 W for female individuals and 160 W for male individuals) in the breath-by-breath mode compared to a stationary metabolic cart (Vyntus CPX). Brehm et al. (2004) showed that M3B overestimated EE by 6.6% at rest and by 2.5% during cycling at 80 W compared to the Douglas bag method. Recently, Van Hooren et al. (2024) evaluated the accuracy of 15 popular cardiopulmonary exercise testing systems in measuring respiratory gas variables, substrate utilization, and EE during simulated and human exercise. In the simulated exercise, M3B overestimated the total EE by 2.29% ± 0.19%, while K5 underestimated the total EE by 6.27% ± 0.19% (Van Hooren et al., 2024). During two rounds of cycling exercises at maximal steady-state intensity, the EE values measured by M3B and Vyntus CPX were 24.0 kcal/min and 23.7 kcal/min, respectively, while the EE values measured by K5 and Vyntus CPX were 16.3 kcal/min and 19.7 kcal/min, respectively (Van Hooren et al., 2024). Although these studies provided valuable insights, they did not directly compare K5 and M3B. In the present study, the results showed substantial differences (639.2 ± 434.8 kcal/day) in EE at rest between K5 and M3B. Similarly, during cycling, the EE measurements from K5 were 0.76–1.18 kcal/min higher across the range of 30 W–60 W compared to M3B values. The discrepancy between previous results and our results may be due to differences in participants, calculation equations, and exercise intensities used, as well as variations in experimental procedures and control conditions across different laboratory settings.

Generally, EE is estimated from respiratory gas exchange measurements using empirically derived formulas for indirect calorimetry (Meyer et al., 2005). Both the devices used in this study appear to utilize the Weir equation to calculate EE (Weir, 1949). Consequently, any differences in EE observed between these devices are likely attributable to factors influencing the accuracy of respiratory gas exchange measurements. These include calibration, masks, ambient sensors, flow sensors, O_2_ and CO_2_ sensors, insufficient sensor warm-up, excessive drift during prolonged use, and other factors (Macfarlane, 2017). Our results demonstrated that K5 consistently recorded higher VE and VO_2_ values than M3B at rest and during cycling. Additionally, while K5 overestimated VCO_2_ compared to M3B at rest, the two devices showed similar VCO_2_ measurements during exercise. The observed differences in VE measurements between K5 and M3B could be attributed to several factors. Although both systems used turbine-based technology, slight variations in turbine design or calibration accuracy might have influenced the results. Even with proper calibration and warm-up, inherent differences in sensor response or data processing algorithms between the two systems could contribute to the discrepancies. Additionally, the use of different facemasks, despite being well-fitting, might have introduced minor leaks or differences in breathing resistance, affecting VE measurements. Furthermore, K5 and M3B both utilize galvanic fuel cells for O_2_ measurement and non-dispersive infrared sensors for CO_2_ measurement; however, K5’s sensors, supplied by City Technology and COSMED, achieve higher precision (±0.02% for O_2_ and ±0.01% for CO_2_) than M3B’s sensors, which are supplied by Teledyne and TreyMed and have a precision of <0.1 vol% (Van Hooren et al., 2024). This difference in precision may result in K5 providing more accurate measurements in high-precision and complex environmental settings. Finally, the weight difference and wearing position between K5 (900 g, worn on the back) and M3B (580 g, worn on the chest) may also be potential factors influencing the results. These factors may increase respiratory resistance, add to the physical burden of movement, disrupt normal breathing patterns, impose psychological stress, and compromise device stability during exercise, all of which may negatively impact measurement accuracy and exercise performance (Macfarlane, 2017).

In the present study, VO_2_ measured by K5 was 35.8% higher at rest and 26.1%–27.7% higher during cycling than that measured by M3B. Leprêtre et al. (2012) reported that the differences in VO_2_ measurements between COSMED K4b2 and CORTEX METAMAX II were 6.3% at rest and 0.1% during maximal exercise; Van Hooren et al. (2024) also found that K5 measured VO_2_ to be 3.27% higher than the reference value, while M3B measured VO_2_ to be only 1.66% higher during maximal steady-state cycling in well-trained individuals. Furthermore, Van Hooren et al. (2024) observed that K5 measured VCO_2_ to be 6.16% higher than the reference value, while M3B measured VCO_2_ to be 0.47% lower; Leprêtre et al. (2012) similarly found that the differences in VCO_2_ measurements between COSMED K4b2 and CORTEX METAMAX II were 18.1% at rest and 26.9% during maximal exercise. Winkert et al. (2020) reported that K5 underestimated VCO_2_ by −12.25% to −0.68% during different stages of cycling compared to the Douglas bag method; however, our study showed no significant difference in VCO_2_ measurements between K5 and M3B during exercise, although K5 overestimated VCO_2_ by 22.7% at rest compared to M3B.

RER is another important variable obtained from the portable metabolic system and can be used to estimate substrate utilization, such as fat oxidation during physical activities (Albouaini et al., 2007). Leprêtre et al. (2012) reported that despite a strong correlation between them, there were substantial differences of up to 15.0% in RER values at rest (0.94 vs 0.81) and during maximal exercise (1.25 vs 0.99) between COSMED K4b2 and CORTEX METAMAX II. Vogler et al. (2010) and Crouter et al. (2019) found that during moderate to vigorous exercise, K5 yielded RER values 0.03% to 0.08% lower than those obtained using the Douglas bag method, while M3B produced higher RER values than both the calibrator and the Douglas bag system. Additionally, Brehm et al. (2004) pointed out that the resting RER value for healthy adult subjects obtained by the M3B was 0.83. These findings are consistent with the results of the current study, which observed significant differences in RER between K5 and M3B. Specifically, the RER values obtained from K5 and M3B were 0.72 ± 0.06 and 0.86 ± 0.05 at rest, respectively. During cycling, the RER values measured by K5 were lower by 0.13–0.18 than those measured by M3B. As RER is calculated as the ratio of VCO_2_ to VO_2_, overestimation or underestimation of either parameter may lead to substantial differences in RER. This is particularly true if the errors in these two parameters differ in direction. In the current study, the higher VO_2_ values measured by K5 led to a lower RER.

Given the frequent use of EE data by untrained female individuals to manage their body weight and health, the present study’s findings hold significant practical implications. For the female individuals without self-reported regular exercise training, cycling at 30 W (4.8 METs), 40 W (5.7 METs), 50 W (6.7 METs), and 60 W (7.9 METs) corresponded to light-to-high relative intensities within their physiological range. During cycling at 30–60 W, the difference in EE measurements between the K5 and M3B systems ranged from 0.76 –1.18 kcal/min across the power range. For instance, an individual might devise a weight-loss regimen involving daily cycling at a 50 W power load for 1 hour. Under this scenario, the daily measured energy discrepancy would amount to approximately 60 kcal (408 kcal vs 348 kcal). Such a discrepancy could create a mismatch between the projected goals and real-world outcomes, thereby undermining the individual’s compliance with the regimen. Meanwhile, the findings of the present study also indicate that it is important to note the differences in EE between different metabolic systems when using them as the criterion methods to develop prediction equations and assess the accuracy of EE estimated by wearable devices. Recently, a systematic review showed that 18 out of 19 studies have utilized portable indirect calorimetry systems as the criterion measure to validate EE measurements of wearable devices, involving four different metabolic systems across these investigations (Parvo Medics TrueOne 2400, Jaeger Oxycon Mobile, COSMED K4b2/K5, and M3B) (Chevance et al., 2022). Results demonstrated discrepancies even for wearable devices of the same brand: certain studies documented an underestimation of EE, whereas others reported an overestimation (Chevance et al., 2022). These conflicting results may be attributable to the use of different reference analyzers.

Several limitations should be considered when interpreting our results. First, our investigation was conducted in a laboratory setting with relatively stable temperature and humidity. Our findings may not be applicable in outdoor or free-living environments characterized by temperature and humidity variations. Second, the present study only included seated rest and low- to high-intensity cycling using a stationary ergometer. Thus, it is unclear what the differences between these two systems are for other intensities and other activity types. Both activity intensity and type influence the results of measurements obtained from metabolic systems (Leprêtre et al., 2012; Macfarlane and Wong, 2012; Macfarlane, 2017; Crouter et al., 2019). Third, no manufacturer staff were involved in calibrating and handling the systems to ensure full compliance with the manufacturer’s guidelines. Even though the researchers were trained and had performed these processes multiple times, operator errors may still occur. Fourth, a significant limitation of this study is the absence of a gold-standard reference method (e.g., the Douglas bag system). This omission precludes a definitive assessment of the absolute accuracy of either device as the observed discrepancies could reflect variations between measurement systems rather than true errors in individual devices. Future research incorporating such gold-standard methods is essential to establish the reliability of these devices in real-world applications.

## 5 Conclusion

K5 and M3B show significant differences in EE measurements during rest and exercise among recreational female individuals, indicating systematic bias in EE measurements between portable metabolic systems. Thus, careful consideration is essential when interpreting the results of wearable-device studies using different automated metabolic systems.

## Data Availability

The raw data supporting the conclusions of this article will be made available by the authors, without undue reservation.

## References

[B1] Albouaini K. Egred M. Alahmar A. Wright D. J. (2007). Cardiopulmonary exercise testing and its application. Postgrad. Med. J. 83, 675–682. 10.1136/hrt.2007.121558 17989266 PMC2734442

[B2] AlSwayied G. Guo H. Rookes T. Frost R. Hamilton F. L. (2022). Assessing the acceptability and effectiveness of mobile-based physical activity interventions for midlife women during menopause: systematic review of the literature. JMIR. Mhealth. Uhealth. 10, e40271. 10.2196/40271 36485026 PMC9789501

[B3] Brehm M.-A. Harlaar J. Groepenhof H. (2004). Validation of the portable VmaxST system for oxygen-uptake measurement. Gait. Posture. 20, 67–73. 10.1016/S0966-6362(03)00097-3 15196523

[B4] Brickwood K.-J. Watson G. O’Brien J. Williams A. D. (2019). Consumer-based wearable activity trackers increase physical activity participation: systematic review and meta-analysis. JMIR. Mhealth. Uhealth. 7, e11819. 10.2196/11819 30977740 PMC6484266

[B5] Caron N. Peyrot N. Caderby T. Verkindt C. Dalleau G. (2016). Energy expenditure in people with diabetes mellitus: a review. Front. Nutr. 3, 56. 10.3389/fnut.2016.00056 28066773 PMC5177618

[B6] Chandrasekaran R. Katthula V. Moustakas E. (2020). Patterns of use and key predictors for the use of wearable health care devices by US adults: insights from a national survey. J. Med. Internet Res. 22, e22443. 10.2196/22443 33064083 PMC7600024

[B7] Chevance G. Golaszewski N. M. Tipton E. Hekler E. B. Buman M. Welk G. J. (2022). Accuracy and precision of energy expenditure, heart rate, and steps measured by combined-sensing fitbits against reference measures: systematic review and meta-analysis. JMIR. Mhealth. Uhealth. 10, e35626. 10.2196/35626 35416777 PMC9047731

[B8] Crouter S. E. LaMunion S. R. Hibbing P. R. Kaplan A. S. Bassett D. R. (2019). Accuracy of the cosmed K5 portable calorimeter. PLoS One 14, e0226290. 10.1371/journal.pone.0226290 31841537 PMC6913985

[B9] Deysel G. Aswegen M. van Kramer M. (2024). Assessing quadriceps strength in patellofemoral pain patients: a study on the reliability and validity of a low-cost strain-gauge for clinical practice. PLoS One 19, e0298570. 10.1371/journal.pone.0298570 38805492 PMC11132478

[B10] El-Gayar O. Elnoshokaty A. (2023). Factors and design features influencing the continued use of wearable devices. J. Healthc. Inf. Res. 7, 359–385. 10.1007/s41666-023-00135-4 PMC10449731 37637719

[B11] Ellingson L. D. Lansing J. E. DeShaw K. J. Peyer K. L. Bai Y. Perez M. (2019). Evaluating motivational interviewing and habit formation to enhance the effect of activity trackers on healthy adults’ activity levels: randomized intervention. JMIR Mhealth Uhealth 7, e10988. 10.2196/10988 30762582 PMC6393778

[B12] Ferguson T. Olds T. Curtis R. Blake H. Crozier A. J. Dankiw K. (2022). Effectiveness of wearable activity trackers to increase physical activity and improve health: a systematic review of systematic reviews and meta-analyses. Lancet. Digital. Health. 4, e615–e626. 10.1016/S2589-7500(22)00111-X 35868813

[B13] Haugen H. A. Chan L.-N. Li F. (2007). Indirect calorimetry: a practical guide for clinicians. Nutr. Clin. Pract. 22, 377–388. 10.1177/0115426507022004377 17644692

[B14] Hill J. O. Wyatt H. R. Peters J. C. (2012). Energy balance and obesity. Circulation 126, 126–132. 10.1161/CIRCULATIONAHA.111.087213 22753534 PMC3401553

[B15] Hopkins W. G. Marshall S. W. Batterham A. M. Hanin J. (2009). Progressive statistics for studies in sports medicine and exercise science. Med. Sci. Sports Exerc 41, 3–13. 10.1249/MSS.0b013e31818cb278 19092709

[B16] Hu R. van Velthoven M. H. Meinert E. (2020). Perspectives of people who are overweight and obese on using wearable technology for weight management: systematic review. JMIR. Mhealth. Uhealth. 8, e12651. 10.2196/12651 31929104 PMC6996738

[B17] Huang S. Dai H. Yu X. Wu X. Wang K. Hu J. (2024). A contactless monitoring system for accurately predicting energy expenditure during treadmill walking based on an ensemble neural network. iScience 27, 109093. 10.1016/j.isci.2024.109093 38375238 PMC10875158

[B18] Jacobs R. E Berg K. Slivka D. R. Noble J. M. (2013). The effect of cadence on cycling efficiency and local tissue oxygenation. J. Strength Cond. Res. 27, 637–642. 10.1519/JSC.0b013e31825dd224 22648142

[B19] Janse D. E. Jonge X. Thompson B. Han A. (2019). Methodological recommendations for menstrual cycle research in sports and exercise. Med. Sci. Sports Exerc 51, 2610–2617. 10.1249/mss.0000000000002073 31246715

[B20] Jonas S. Phillips E. M. (2012). ACSM’s exercise is medicineTM: a clinician’s guide to exercise prescription. Pennsylvania, Philadelphia: Lippincott Williams and Wilkins.

[B21] Leprêtre P. M. Weissland T. Paton C. Jeanne M. Delannaud S. Ahmaidi S. (2012). Comparison of 2 portable respiratory gas analysers. Int. J. Sports Med. 33, 728–733. 10.1055/s-0031-1301316 22562743

[B22] Lindgren T. Hooper J. Fukuoka Y. (2019). Perceptions and experiences of women participating in a digital technology-based physical activity intervention (the mPED trial): qualitative study. JMIR. Public. Health. Surveill. 5, e13570. 10.2196/13570 31859677 PMC6942195

[B23] Liu H. Li Q. Li Y. Wang Y. Huang Y. Bao D. (2022). Concurrent validity of the combined HRV/ACC sensor and physical activity diary when monitoring physical activity in university students during free-living days. Front. Public Health 10, 950074. 10.3389/fpubh.2022.950074 36159256 PMC9496871

[B24] Macfarlane D. J. (2017). Open-circuit respirometry: a historical review of portable gas analysis systems. Eur. J. Appl. Physiol. 117, 2369–2386. 10.1007/s00421-017-3716-8 29043499

[B25] Macfarlane D. J. Wong P. (2012). Validity, reliability and stability of the portable cortex metamax 3B gas analysis system. Eur. J. Appl. Physiol. 112, 2539–2547. 10.1007/s00421-011-2230-7 22075643 PMC3371330

[B26] Meyer T. Davison R. C. R. Kindermann W. (2005). Ambulatory gas exchange measurements--current status and future options. Int. J. Sports Med. 26 (Suppl 1), S19–S27. 10.1055/s-2004-830507 15702452

[B27] Nuss K. Moore K. Nelson T. Li K. (2021). Effects of motivational interviewing and wearable fitness trackers on motivation and physical activity: a systematic review. Am. J. Health Promot 35, 226–235. 10.1177/0890117120939030 32662277

[B28] O’Driscoll R. Turicchi J. Beaulieu K. Scott S. Matu J. Deighton K. (2020). How well do activity monitors estimate energy expenditure? A systematic review and meta-analysis of the validity of current technologies. Br. J. Sports Med. 54, 332–340. 10.1136/bjsports-2018-099643 30194221

[B29] Overstreet B. S. Bassett D. R. Crouter S. E. Rider B. C. Parr B. B. (2017). Portable open-circuit spirometry systems. J. Sports Med. Phys. Fit. 57, 227–237. 10.23736/S0022-4707.16.06049-7 26861831

[B30] Perez-Suarez I. Martin-Rincon M. Gonzalez-Henriquez J. J. Fezzardi C. Perez-Regalado S. Galvan-Alvarez V. (2018). Accuracy and precision of the COSMED K5 portable analyser. Front. Physiol. 9, 1764. 10.3389/fphys.2018.01764 30622475 PMC6308190

[B31] Shin G. D. Jeong W. Lee H.-E. (2023). Factors affecting female college students’ intention to use digital technology in wearable devices to stimulate health monitoring. Heliyon 9, e18118. 10.1016/j.heliyon.2023.e18118 37539275 PMC10395341

[B32] Thun E. Bjorvatn B. Flo E. Harris A. Pallesen S. (2015). Sleep, circadian rhythms, and athletic performance. Sleep. Med. Rev. 23, 1–9. 10.1016/j.smrv.2014.11.003 25645125

[B33] Van Hooren B. Souren T. Bongers B. C. (2024). Accuracy of respiratory gas variables, substrate, and energy use from 15 CPET systems during simulated and human exercise. Scand. J. Med. Sci. Sports 34, e14490. 10.1111/sms.14490 37697640

[B34] Vogler A. J. Rice A. J. Gore C. J. (2010). Validity and reliability of the cortex MetaMax3B portable metabolic system. J. Sports Sci. 28, 733–742. 10.1080/02640410903582776 20419553

[B35] Weir J. B. (1949). New methods for calculating metabolic rate with special reference to protein metabolism. J. Physiol. 109, 1–9. 10.1113/jphysiol.1949.sp004363 15394301 PMC1392602

[B36] Winkert K. Kirsten J. Dreyhaupt J. Steinacker J. M. Treff G. (2020). The COSMED K5 in breath-by-breath and mixing chamber mode at low to high intensities. Med. Sci. Sports Exerc 52 (5), 1153–1162. 10.1249/MSS.0000000000002241 31895296

